# IOC Olympian Health Cohort: a study protocol for a 15-year, prospective, Olympian health study across Summer and Winter Olympic sports

**DOI:** 10.1136/bmjsem-2024-002061

**Published:** 2024-06-06

**Authors:** Debbie Palmer, Torbjorn Soligard, Gwen Fernandes, Dave Collins, Niall Elliott, Paul Kelly, Iain Murray, Lars Engbretsen

**Affiliations:** 1 Edinburgh Sports Medicine Research Network, Institute for Sport, PE and Health Sciences, The University of Edinburgh, Edinburgh, UK; 2 UK Collaborating Centre on Injury and Illness Prevention in Sport (UKCCIIS), The University of Edinburgh, Edinburgh, UK; 3 IOC Medical and Scientific Commission, Lausanne, Switzerland; 4 Academic Rheumatology, School of Medicine, University of Nottingham, Nottingham, UK; 5 Grey Matters Performance, London, UK; 6 Sports & Exercise Medicine, Sportscotland Institute of Sport, Stirling, UK; 7 Physical Activity for Health Research Centre, The University of Edinburgh, Edinburgh, UK; 8 Edinburgh Sports Medicine Research Network, Institute for Sport, PE and Health Sciences, Edinburgh Orthopaedics, Edinburgh, UK; 9 Department of Sports Medicine, Norwegian School of Sports Sciences, Oslo, Norway

**Keywords:** Injury, Illness, Athlete, Prevention

## Abstract

Prevention of sports injury and illness and protection of athlete health are key mandates of the IOC. Methodological limitations in Olympic Games surveillance and retired Olympian studies mean there are gaps in the available evidence on Olympian health and the varied challenges occurring at different stages throughout an athlete’s career. This (protocol) paper describes the methods for implementation of the IOC Olympian Health Cohort. The study aims to establish a longitudinal cohort of current Olympians and follow them prospectively (around 15 years) throughout their Olympic careers and retirement. The study will use participants who have completed self-report questionnaires. Olympians will be recruited after each Summer and Winter Olympic Games, and all National Olympic Committee (NOC) athletes aged 16 years or older are eligible. The first phase included the Tokyo 2020/2021 and Beijing 2022 Olympians, with the study promoted via IOC platforms, Athlete365 and NOCs. Questionnaires include baseline demographics, sports exposure and history of injuries and illnesses impacting the athlete’s ability to continue to train and/or compete for at least 2 weeks. Questions also address retirement from sports, musculoskeletal, mental and general health, and quality of life measures. This protocol describes the methods for the 15-year global IOC Olympian Health Cohort Study, from participant recruitment to the development and distribution of the study questionnaire. This protocol will be updated to report future changes in the study’s conduct or questionnaire content. These data will help identify risk factors and inform risk-reduction strategies. The ultimate goal is to protect the health of all athletes during their careers and retirement.

**WHAT IS ALREADY KNOWN ON THIS TOPIC**

- Elite sport participation is associated with an increased risk of injury, illness and other health-related issues. Between data captured during Olympic Games injury and illness surveillance studies and cross-sectional retired Olympian studies, there are gaps in the evidence on Olympian health and what happens to athletes throughout their sporting careers.

**WHAT THIS STUDY ADDS**

- This study will be the first global study surveying elite athletes prospectively on various injuries and illnesses and other health and well-being challenges throughout different stages of their sports careers and into retirement. It will allow the identification of risk factors associated with common health-related issues athletes experience, as well as investigation across geographical areas, sports settings and contexts, and ad hoc exploration of new and emerging issues.

**HOW THIS STUDY MIGHT AFFECT RESEARCH, PRACTICE OR POLICY**

- Findings from this study will be shared with key stakeholders to help researchers, sports federations, coaches and support staff, and athletes understand risk factors associated with athlete health and well-being challenges more accurately. The fundamental aim is to better inform prevention strategies to reduce their occurrence and limit the progression of more serious outcomes.

## Background

Sports injury, illness prevention and the protection of athlete health are key mandates of the IOC.^1^ While exercise and sport participation confer several health benefits, sport at the elite level can also carry an associated increased risk of health problems, such as musculoskeletal injury, challenges to mental well-being and illness. Continued robust research on athlete health problems, alongside greater international community engagement, is needed to help inform risk-reduction strategies in elite sport and improve athlete health and well-being.^2^

Much is known about the occurrence and nature of elite athlete injuries and illnesses during major sporting events, such as the Olympic Games, World Cup tournaments within football (soccer) and rugby, and athletics World Championships.^3–5^ At the most recent Tokyo 2020/2021 Summer and Beijing 2022 Winter Olympic Games, IOC in-games surveillance studies reported that 9–10% of athletes were injured, and around 4% were ill.^6 7^ Outside of major tournaments, however, no international studies prospectively follow elite athlete injuries or illness during the whole of their sporting careers.

There is a growing body of knowledge on what happens to elite athletes when they retire from sport, for example, higher rates of pain and post-traumatic osteoarthritis (OA) but better self-reported general health in later years compared with the general population.^8^ In a recent retired Olympian study, 63% of Olympians reported having had a significant injury at some point during their career.^9^ Significant joint injury is a risk factor for the development of OA, and there is evidence from football, Rugby Union and Olympic sports reporting an association between joint injury and ongoing pain and the development and progression of OA in retired athletes.^10–14^

### Limitations to current knowledge

Athlete injury and illness surveillance studies during the Olympic Games continue to provide important ongoing information. However, these data are limited to data capture during a 3-week window, once every 4 years.^6 7^ Conversely, most retired athlete health studies are cross-sectional, and there are limitations to these studies regarding recall bias due to the retrospective nature of injury and health history questions.^9^ So while these studies provide important new knowledge, there are gaps in our understanding of Olympian health and what happens to these athletes during their careers outside of major events like the Olympic Games (reported injury rates 9–10%) and as they enter retirement (reported injury rate 63%).^6 7 9^

### New and emerging issues

In addition to musculoskeletal health, there have been several other high-profile athlete health and well-being issues emerging over the last few years, for example, sport-related concussions, harassment and abuse in high-performance sports, and elite athlete mental health. These factors often coexist alongside musculoskeletal and other physical challenges, creating a potential negative cocktail with which the athlete must cope. Recent consensus statements have focused on some of these issues.^15–17^ The IOC consensus on harassment and abuse in sport suggests a multiagency approach including athletes for effective prevention,^16^ and the consensus on mental health in elite athletes advocates management strategies be focused on addressing all contributors to mental health symptoms in athletes.^17 18^

To understand the magnitude of injury and illness and other health and well-being issues in Olympians (including the interaction between physical health and other health and well-being measures), and to be able to ascertain more accurate risk factors for short and long-term athlete health and well-being, prospective monitoring of Olympians throughout their competitive careers is needed.^19^ The ability to survey current Olympic athletes on these and other issues will help provide objective athlete-centred information, which is a strength of this study design. This information could then inform evidence-based targeted interventions to help mitigate some of the negative outcomes associated with elite sports participation. It will also allow for monitoring changes over time to assess the effectiveness of those risk-reduction strategies.

The aim of this paper is, therefore, to describe the methods for implementation of the new IOC long-term prospective Olympian Health Cohort, including study objectives, participant selection, recruitment, as well as development, refinement and distribution of the study questionnaire, and cohort maintenance.

## Methods

### IOC cohort aims and objectives

The IOC Olympian Health Cohort Study aims to recruit and establish a cohort of current Olympians competing in the Summer and Winter Olympic Games to follow them prospectively throughout their Olympic careers and into retirement. Setting up this Olympian database will enable more timely surveying of musculoskeletal and general health measures at different stages of the athletes’ careers and as they transition out of sport and into retirement. This global cohort will allow the investigation of differences across geographical settings and contexts. It will also allow ad hoc exploration of topical new and emerging issues in elite athlete health, enabling us to track these conditions from precursive factors to long-term impact.

Overall study objectives are to: (1) recruit a representative sample of recent Summer and Winter Olympians across different geographical areas and sports; (2) prospectively follow and survey the Olympian cohort (target follow-up time of 15 years) to understand musculoskeletal and general health and well-being issues more accurately (a) during their careers, (b) as they transition out of sport and (c) in retirement; (3) provide early identification of risk factors for common current known health issues (eg, pain and OA), and early identification of risk factors for new and emerging health and well-being issues (eg, mental health, concussion, harassment and abuse in sport, recovery from COVID-19); and (4) identify the interaction between athlete physical health, mental health and other health and well-being issues.

### Olympian cohort

The study will be a prospective longitudinal cohort of current Summer and Winter Olympians, using participant-completed questionnaire data collection.

All National Olympic Committee (NOC) athletes aged 16 years or older who have competed at Summer or Winter Olympic Games (Olympians) will be eligible and invited to participate in this study. The first phase of the study included athletes who participated in the Tokyo 2020/2021 and Beijing 2022 Olympic Games. Athletes who will compete at future Summer and Winter Olympic Games, for example, Paris 2024, Milano/Cortina 2026 Olympic Games, will also be invited to participate in the study. Olympians will be followed up prospectively for the next 15 years. The cohort will consist of current Olympians and include some newly retiring Olympians (ie, those who retired after their Olympic Games participation). Over time, Olympians who stop competing and transition out of sport will be deemed retired Olympians.

### Cohort recruitment and consent

Olympians will be recruited immediately after each Olympic Games period. Phase one began in August 2022 and finished in July 2023. It included those who had competed at the Tokyo 2020 Summer Olympic Games and Beijing 2022 Winter Olympic Games. Based on participation rates across both games, this equates to around 11 000 eligible athletes at each Summer Games and 3000 athletes at each Winter Games.

Study promotional materials will be created, including a video comprising well-known current and retired Olympians across different sports and geographical areas. Materials, including study information and recruitment details, will be promoted via IOC platforms, the Athlete365 network, Olympic Athlete Hub, Athlete Learning Gateway and the World Olympians Association OLY databases. Recruitment will also occur through NOCs and National Olympians Associations (NOAs) via email, Facebook and other social media. Questionnaires will be distributed through contact emails from recruitment phases using embedded questionnaire survey links on a secure electronic platform (Qualtrics). Study information will be provided to participants before each survey, and detailed participant information sheets (PIS) will be embedded at the start of each questionnaire. In each PIS and on each questionnaire, it will be outlined that on completion and return of the questionnaire, participants will be deemed to have consented to their information being used for the study.

### Cohort maintenance

Various cohort maintenance activities will occur to maintain the cohort and reduce participant dropout between study phases. Strategies employed include a dedicated study participant website with resources, news and information on publications and other materials related to the study: https://olympians.ed.ac.uk/. There will be a biennial study newsletter sent directly to Olympians. New questionnaire measures (ie, special topic areas) will be introduced, and the purpose will be outlined in detail to the cohort. Ad hoc news at other time points will coincide with Olympian recruitment phases, paper publications and other study media articles. There will also be a prize draw at each survey time point for all those who return a completed questionnaire.

### Questionnaire

The questionnaire was developed in conjunction and collaboration with the assembled core research group, which includes members of the IOC injury prevention research group at the Medical and Scientific Department. Additional expertise will be sought and included to inform future questionnaire content and adaptations around current and new topical issues (eg, abuse and harassment in sports) as and when they arise.

1. Demographics: baseline questions include contact information confirmation, age, sex, education and occupation (if relevant), stature, Olympic sport and years of participation. For those transitioning out of sport, questions will include post-sports occupations. Updated relevant information will be collected at each time point.

2a. Injury and illness history: injury and illness data recording will be completed in line with the consensus of the IOC methodology and previous Olympian health studies.^9 20^ Injury recording will include injury location, type of injury, severity (days) and cause of injury, including recurrent injuries and residual symptoms (eg, pain and stiffness). The affected system, main symptoms, cause and severity will also be recorded for illnesses. A significant Olympic career sport-related injury/illness definition will be ‘any injury/illness that occurred during training or competition that impacted the athlete’s ability to continue to train and/or compete for most days for at least 2 weeks’. Injuries and illnesses occurring outside of Olympic sports activities will also be recorded. They will be defined as ‘any injury or illness that impacted the athlete physically for most days for at least 2 weeks’. Injury/illness severity will be defined by the estimated days between the injury/illness and the athlete’s full return to sports participation. Ongoing exposure to training, competition and other athlete behaviours and treatments (eg, medication use, surgery, joint injection) during injury and illness will also be recorded.^9 12 13^

**Figure 1:**
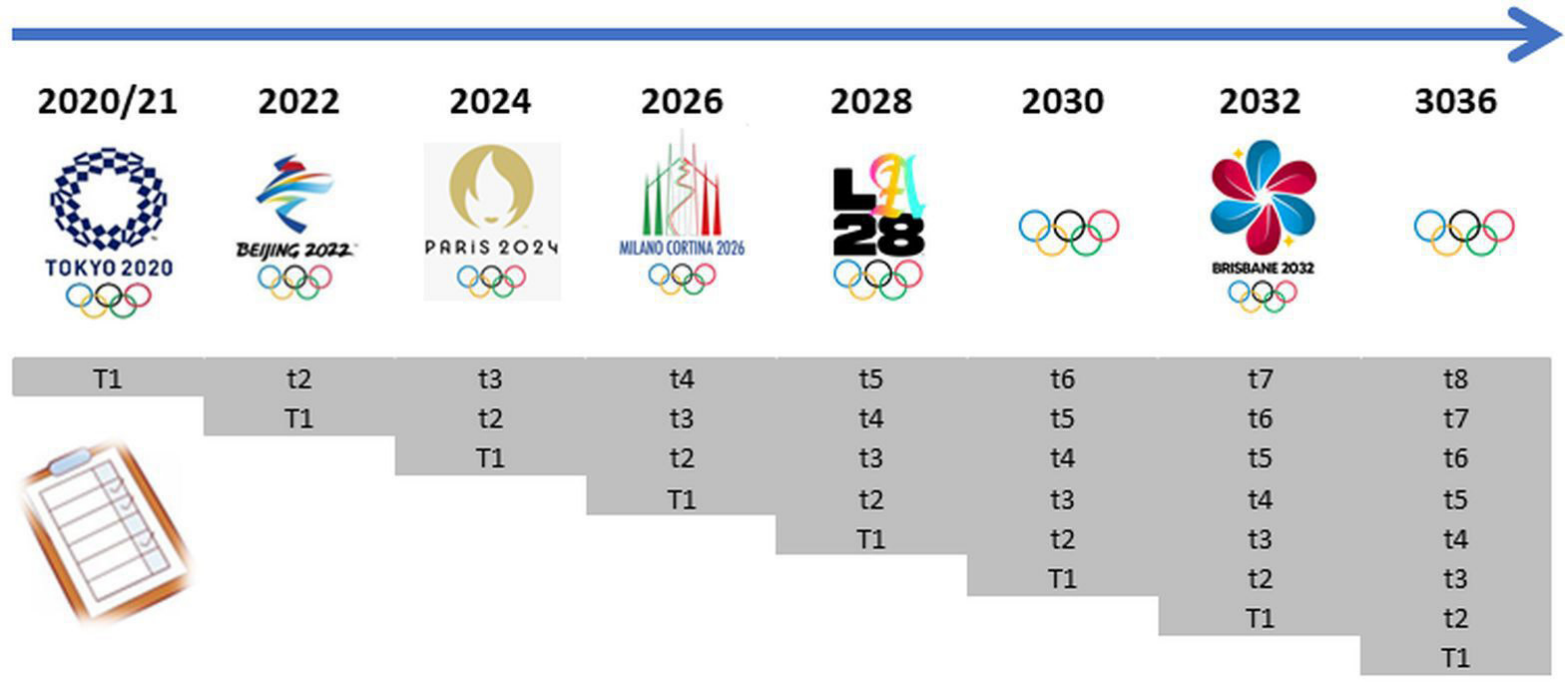
Participant questionnaire timeline.

Questionnaires will ask newly recruited participants at each initial phase (T1—figure 1) to record all of their Olympic sport and other (non-Olympic sport, non-sport) related injuries and illnesses over the preceding 4 years, equating their full Olympic cycle (T1—figure 1). Subsequent biennial surveys of the current cohort will only ask participants to report their injuries and illnesses from the previous 2 years (t2–t8, figure 1).

2b. Sport exposure: time spent in competition and training will be recorded as exposure to the risk environment based on athlete estimations. Competitions will be recorded by level (eg, national, international), number per year and estimated time in hours. Training refers to any/all Olympic sport-specific activities and will be recorded by the type and number of sessions and estimated time in hours.^9 20^

3. Retirement from sport: a retired Olympian is defined as ‘an Olympian who considered themselves retired from Olympic and international competition, who no longer intended to compete in any upcoming Summer or Winter Olympic Games’. Questions around Olympians’ retirement from competitive sport will include the nature of retirement (eg, injury/illness related, planned, unplanned), mental health status, perceived challenge and progress measures, and current and future career/education plans and level of support during transition out of sport, for example, career advisor support.

4. General health and quality of life: activity assessment questions will include the International Physical Activity Questionnaire; general health measures will include alcohol and tobacco use, current disease states (eg, heart disease, cancer) and EQ5D5L General Health-related Quality of life Survey containing five dimensions—(1) mobility (2) self-care (3) usual activities, (4) pain/discomfort and (5) anxiety/depression, plus a general health perception score.^21^

5. Well-being, mental health and resilience: in addition to EQ5D5L item 5, mental health measures will include self-report Patient Health Questionnaire (PHQ-9) depression screening, providing demarcation of mild, moderate, moderately severe and severe, including normative data.^22 23^ Experiences of interpersonal violence as a measure of abuse and harassment will be recorded.^24^ In the ‘absence of mental ill-being’, general psychological well-being measures will include resilience and coping and psychological characteristics and skills (eg, the Nicholson McBride Resilience Questionnaire).^25–27^ Overall measures of ‘perceived challenge and progress’ will be combined to assess the impacts of physical and mental chronic stressors on athletes over time. As athletes retire, these perceived progress measures will change focus from sport to general life and post-sport career activities.

6. Musculoskeletal health: current pain (widespread and by individual location), musculoskeletal dysfunction and stiffness (including linked locations from injuries in section 2a) will be recorded, alongside diagnosis, and markers and risk factors for OA. Questions will also be asked about participants’ history of surgery, including joint surgery.^10–13^

7. Special topic areas: more detailed questionnaire sections on specific topic areas will be developed when required and included in ‘long-form’ questionnaires. Topics may include, for example, concussion, cognitive health, overtraining (physical and mental burden), metabolomics/genomics, abuse/harassment in sport, female athlete health and relative energy deficiency. In addition, there may be limited ad hoc questionnaire time points for time-sensitive, critical issues. The intention is to track factors as precursors or antecedents to later issues. Targeted ad hoc survey use will be tightly controlled and limited to not ‘fatigue’ the cohort. In addition, the purpose of each measure will be clarified to the cohort to promote active involvement and adherence.

Indicative serial questionnaire time points are presented in figure 1. Surveys will include the ‘T1’ baseline questionnaire for all new study participants and repeated time point follow-up questionnaires (t) every 2 years.

### Patient and public involvement

Questionnaire(s) will go through patient and public involvement piloting with a like-for-like cohort of elite athletes. Feedback will be sought to allow for the review, adaptation and finalisation of the questionnaire tool. As part of each survey, participants will also be provided an opportunity in an open-ended free text box to highlight topic areas they feel are important to them and that were missed. The research team will use this feedback to inform and refine the survey questions for the next recruitment phase.

### Outcome variables and planned analysis

The primary outcomes are the prevalence of injury and illness, aetiology and associated risk factors during an Olympic career (and beyond). Secondary outcomes include injury and illness treatments and behaviours, prevalence and risk factors for pain, ongoing musculoskeletal dysfunction (including OA) and risk factors for mental health, cognitive health and other health and well-being outcomes.

Descriptive statistics will be presented as the mean and SD for numerical variables and frequencies (proportion) for categorical variables. Prevalence will be calculated based on the number of athletes with the health problem divided by the total number of athletes and presented as percentage (%) with 95% CIs. Differences between groups for numerical variables will be analysed by t-test or Mann-Whitney where appropriate, and for categorical variables, using the χ^2^ test.^28^ Relative risk (with 95% CI) between groups will be calculated using the Poisson regression model, assuming constant hazard per group and adjusting for sport, sex, age or group size where appropriate. Study analysis will allow cross-sectional and longitudinal analysis of changes over time.

### Confidentiality and study governance

The University of Edinburgh is the study sponsor and will oversee all aspects of research governance. Participant data will be de-identified, and a universal ID will be used at all stages of the study. All data will be treated confidentially, ensuring anonymity at all times.

There will be no significant risks to Olympians associated with their participation in the study, but some questions around injury, illness occurrence or mental health may be sensitive. Purposefully, no survey questions will be mandatory, and participants will be free to omit any questions they do not wish to answer. Help resources are provided alongside the PHQ-9 within the survey and again at the end. This includes links to the IOC Athlete365 health and safeguarding team and their website (https://olympics.com/athlete365/what-we-do/integrity/safe-sport/).

Participants will be informed that they can discontinue the study at any time. Each individual will be assigned a unique and linked study ID number linked to their email address in a separate file. Linking individual participant data will only occur at repeat questionnaire time points. Individuals’ unique study ID numbers will be used at all times, within data storage and data analysis, to ensure anonymity and confidentiality throughout all stages of the study. This is in adherence to the University of Edinburgh research and information governance protocol, including robust data storage mechanisms for research studies.

## Discussion

In the recent IOC consensus on methods for recording and reporting injury and illness, the authors strongly encourage researchers to publish their study protocols in an open-access format before study completion.^20^ Therefore, the purpose of this paper was to describe the methods for the 15-year prospective IOC Olympian Health Cohort Study, from participant recruitment to the development and distribution of the study questionnaire. Going forward, this protocol document will be updated to report on changes in study conduct or amendments to study questionnaire content.

The study team has a strong track record of producing high-impact research. It has diverse research and clinical backgrounds (from injury epidemiology, psychology, public health, OA, orthopaedics and sports medicine) and career stages (early, mid and late). We anticipate at least two peer-reviewed journal articles at each phase of study recruitment: (1) describing the recruited cohort, including Olympic career, injury, illness, general health and mental health measures; and (2) the interactions between Olympic career health issues and risk factors such as injury occurrence and behaviours during injury, and ongoing pain, musculoskeletal dysfunction and mental health (for example).

With help and collaboration with the IOC and NOCs, our study aims to recruit and establish a global cohort of Summer and Winter Olympians across multiple Olympic Games cycles. The study will allow prospective surveying and monitoring of a range of athlete health issues across different stages of the athletes’ careers, helping more accurately identify risk factors associated with some of the common health-related issues athletes experience. It will allow investigation across different geographical areas, sports settings and contexts, and ad hoc exploration of new and emerging athlete health and well-being issues. For example, concussions in sports, athlete harassment and abuse, and athlete mental health. Early identification and better understanding of some of the causes of these current and emerging issues may help limit the progression of more serious disease outcomes.

### Knowledge mobilisation and impact plan

Results will be shared with Olympians recruited to the cohort and key stakeholders and funders, including the IOC, Athlete365, International Federations, NOCs and NOAs. Findings will also be shared at scientific meetings and conferences. These data will help researchers, sports organisations, governing bodies, coaches, support staff and athletes themselves understand more accurately some of the health and well-being challenges they experience during their careers. In turn, these data will be used to help better inform prevention strategies while monitoring any changes over time to assess the effectiveness of those prevention interventions.

### Limitations and ethical considerations

While the current study hopes to negate some of the existing methodological challenges associated with in-games injury and illness surveillance studies and historical retired athlete studies, there will be limitations associated with the present study. Data numbers across some categories, that is, across multiple Summer and Winter Olympic sports, may be small, and hence, early study results may be affected by sparse data bias.^29^ Inherent with self-report survey studies, there may be a self-selection bias in our study, whereby Olympians experiencing more health-related issues may be more likely to participate and take part in the survey. A prize draw will be included at each phase of the study, and promotional materials will be developed using athlete advocates from a range of Summer and Winter Olympic sports and different geographical areas to incentivise and motivate a wider demographic of Olympians to participate. There remains a retrospective component to injury and illness-related questions where Olympians will initially report injuries and illness over the preceding 4 years and then subsequently every 2 years. A significant 2-week definition of injury and illness will be used to negate associated recall bias. This will allow for a better recollection of important injury or illness issues; however, it will mean that less severe injuries and illnesses (>2-week duration) will not be reported.

Finally, it is understood that some survey questions may be sensitive in nature. For this reason, survey questions are not mandatory, and participants can miss any questions they do not wish to answer. Additional resources and sources of support will be embedded within the surveys and sign-posted to participants at relevant points (eg, mental health questionnaire sections) and again at the end of the surveys to participants.

## Conclusions

Sport participation at the elite level is associated with increased risks of injury, illness and other health problems. Significant gaps exist in the available evidence and, hence, in our understanding of what happens to elite athletes throughout their competitive careers. The IOC Olympian Health Cohort will be the largest prospective study of elite athlete health and the first study to recruit participants globally across multiple Summer and Winter Olympic sports. Prospective monitoring of Olympians will help us understand the magnitude of the problem and provide new knowledge on risks associated with various health-related issues experienced by elite athletes. These data will help inform risk-reduction strategies and allow objective assessment of the impact of those interventions. The ultimate goal is to protect the health of all athletes, both during their careers and through retirement from sport.

## Data Availability

All data relevant to the study are included in the article or uploaded as supplemental information.
